# Influence de la numération leucocytaire sur la densité parasitaire dans le paludisme simple chez les enfants de 6 à 59 mois au Bénin

**DOI:** 10.48327/mtsi.v3i2.2023.321

**Published:** 2023-05-31

**Authors:** Tatiana BAGLO, Alban Gildas Comlan ZOHOUN, Lutécia ZOHOUN, Antoine SIANOU, Dorothée KINDÉ GAZARD

**Affiliations:** 1 Laboratoire d'hématologie, Centre national hospitalier universitaire Hubert Koutoukou Maga (CNHU-HKM), Cotonou, Bénin; 2 Faculté des sciences de la santé de Cotonou, Université d'Abomey-Calavi, Bénin; 3 Programme national de lutte contre le paludisme (PNLP-Bénin)

**Keywords:** Paludisme, Densité parasitaire, Numération leucocytaire, Frottis sanguin, Goutte épaisse, Bénin, Afrique subsaharienne, Malaria, Parasite density, Leukocyte count, Blood smear, Thick drop, Benin, Sub-Saharan Africa

## Abstract

**Contexte:**

Un volet essentiel de la prise en charge du paludisme est le diagnostic biologique, dont la technique de référence est la goutte épaisse (GE) associée au calcul de la densité parasitaire (DP). Cette dernière est déterminée sur la base du nombre de parasites comptés dans un champ microscopique par rapport à un nombre donné de leucocytes. Sachant que le nombre de globules blancs chez un patient varie en fonction de l’état physiologique et du contexte clinique, notre étude avait pour objectif de déterminer l'impact de la numération leucocytaire sur le calcul de la densité parasitaire dans les cas de paludisme simple.

**Méthode:**

L’étude était transversale à visée analytique et s’était déroulée dans 2 hôpitaux du Bénin. Elle a porté sur une population de 476 enfants âgés de 6 à 59 mois inclus pour une fièvre. La goutte épaisse et l'hémogramme ont été systématiquement réalisés chez tous les enfants inclus. La densité parasitaire a été calculée selon 3 méthodes en utilisant d'abord un nombre pondéré de 6 000/mm^3^ de leucocytes recommandé par le Programme national de lutte contre le paludisme (PNLP) du Bénin, puis un nombre de leucocytes à 8 000/mm^3^ recommandé par l'Organisation mondiale de la Santé et enfin le nombre réel de leucocytes du patient obtenu sur l'hémogramme.

**Résultats:**

À l'issue de notre étude, 313 enfants soit 65,8 % de notre population d’étude avait une GE positive. Le nombre de leucocytes chez ces derniers était en moyenne de 11 580/ mm^3^. En utilisant successivement le nombre moyen de 6 000 leucocytes/mm^3^ proposé par le PNLP-Bénin et celui de 8 000 leucocytes/mm^3^ proposé par l'OMS, la moyenne des densités parasitaires était respectivement de 47 943 et de 63 936 trophozoïtes/µl contre 92 290 trophozoïtes/µl lorsque le nombre réel de leucocytes des patients était utilisé pour le calcul de la DP. En utilisant une moyenne de 6 000 leucocytes/mm^3^ pour le calcul de la DP, 60 % des DP calculées avaient été sous-estimées et 6 % étaient surestimées. L'utilisation de la moyenne de 8 000 leucocytes/mm^3^ avait entraîné une sous-estimation de la DP dans 49 % des cas et sa surestimation dans 15 % de cas. La différence entre les trois méthodes de calcul a été considérée comme significative.

**Conclusion:**

L'utilisation des coefficients 6 000 ou 8 000 pour l'estimation de la parasitémie pourrait entraîner une sous-estimation significative de la charge parasitaire.

## Abstract (en)

**Background:**

For many years, the treatment of malaria was based on clinical presumptive diagnosis, making its differential diagnosis with other causes of hyperthermia difficult. This drug pressure has led to the emergence of *Plasmodium* strains resistant to the most commonly used antimalarial drugs. This is why in 2004, the health authorities decided to revise the policy of malaria management by adopting a new strategy based on the rational use of artemisininbased combination therapies after the biological confirmation of suspected malaria cases. The biological diagnosis is an essential part of malaria management. The gold standard technique for diagnosis is the thick drop combined with the calculation of parasite density (PD), which is determined on the basis of the number of parasites counted in a microscopic field against a proposed standard number of leukocytes. The number of leukocytes used to calculate the parasite density should ideally be the actual number of leukocytes in the patient per cubic millimetre of blood. However, in the absence of the availability of a blood count at the time of the thick drop, an average number of 8 000 leukocytes/mm^3^ was used by the World Health Organisation (WHO) to estimate the parasite density. Nonetheless, in Benin the average number of leukocytes adopted by the National Malaria Control Programme (PNLP) is 6 000/mm^3^. The aim of our study was to determine the impact of the leukocyte count on the calculation of the parasite density in cases of uncomplicated malaria.

**Method:**

The study was a cross-sectional study with an analytical aim and took place in 2 hospitals in Benin, the Klouékanmey zone hospital in the south of Benin and the Djougou health centre in the north. It involved a population of 476 children aged between 6 and 59 months who were seen in consultation and in whom the clinical diagnosis of simple *Plasmodium falciparum* malaria was suspected. Children aged between 6 and 59 months, weighing at least 5 kg, with an axillary temperature ≥ 37.5°C at the time of consultation or a history of fever in the last 24 hours or other symptoms pointing to the diagnosis of malaria were included. Infestation was mono-specific for *Plasmodium falciparum.* Informed consent was required from the child's parents or guardian. The criteria for non-inclusion in our study were the presence of at least one sign of malaria severity, signs of severe malnutrition or a febrile state related to underlying infectious diseases other than malaria. Thick blood count and haemogram were systematically performed in all included children. Parasite density was calculated according to 3 methods, first using a weighted leukocyte count of 6 000/mm^3^ recommended by the Benin National Malaria Control Programme (PNLP), then a leukocyte count of 8 000/mm^3^ recommended by the World Health Organisation and finally the patient's actual leukocyte count obtained from the blood count. It should be noted that these different samples were respectively taken on the day of inclusion in compliance with the conditions of the pre-analytical phase in force in our medical biology laboratory.

**Results:**

At the end of our study, 313 children, i.e. 65.76% of our study population had a positive white blood cell count with a positivity rate of 62.14% in Djougou, i.e. 174 children, and 70.9% in Klouékanmey, i.e. 139 children. The average leukocyte count in these children was 11,580/mm^3^. Among them, 205 children had an abnormal white blood cell count, i.e. 17 cases of leukopenia (5.43%) and 188 cases of hyperleukocytosis (60.06%). Using successively the average number of 6 000 leukocytes/mm^3^ proposed by the Benin PNLP and that of 8 000 leukocytes/mm^3^ proposed by the WHO, the average parasite densities were respectively 47,943 and 63,936 trophozoïtes/µl against 92,290 trophozoïtes/µl when the real number of leukocytes of the patients was used for the calculation of the PD. By using an average of 6 000 leukocytes/mm^3^ for PD calculation, 60% of the calculated PDs were underestimated and 6% were overestimated. Using an average of 8 000 leukocytes/mm^3^ resulted in 49% of PD being underestimated and 15% being overestimated. The difference between the three calculation methods was considered statistically significant (p value <0.05).

**Conclusion:**

The use of 6 000 or 8 000 coefficients for the estimation of parasitaemia could lead to a significant underestimation of the parasite load.

## Introduction

Le paludisme est une érythrocytopathie parasitaire fébrile due à des hématozoaires du genre *Plasmodium,* transmis par la piqûre d'un moustique, l'anophèle femelle. En zone d'endémie, ce sont les enfants âgés de moins de 5 ans, les femmes enceintes et les sujets âgés qui payent le plus lourd tribut, et chez qui la mortalité reste la plus élevée. Au Bénin, en 2015, 1747 décès liés au paludisme ont été enregistrés [1, 9].

Pendant de nombreuses années, le traitement du paludisme était basé sur le diagnostic présomptif sans confirmation biologique, rendant difficile son diagnostic différentiel avec les autres causes d'hyperthermie. Cette pression médicamenteuse a induit l’émergence des souches de *Plasmodium* résistantes aux antipaludiques les plus utilisés tels que la chloroquine et la sulfadoxine-pyriméthamine [11]. Plusieurs auteurs ont rapporté cette chloroquino-résistance au Bénin [2, 3, 12]. Face à ce constat, la politique de prise en charge du paludisme a été révisée avec l'adoption d'une nouvelle stratégie basée sur l'utilisation rationnelle des combinaisons thérapeutiques à base d'artémisinine (CTA) après confirmation biologique des cas suspects d'accès palustre [10]. Divers moyens permettent de diagnostiquer le paludisme, notamment le test de diagnostic rapide (TDR), le frottis sanguin (FS) et la goutte épaisse (GE) qui est le Gold Standard [13]. En effet, la GE permet le calcul de la densité parasitaire en décomptant le nombre de parasites asexués par rapport à un nombre de globules blancs. Le nombre de leucocytes devrait idéalement être le nombre réel de leucocytes du patient par millimètre cube de sang [5]. Mais en absence de la disponibilité systématique de l'hémogramme, un nombre pondéré de 8 000 leucocytes/mm^3^ a été retenu par l'OMS pour estimer la densité parasitaire [8]. Au Bénin, le nombre moyen de leucocytes adopté par le Programme national de lutte contre le paludisme (PNLP) est de 6 000/mm^3^ [10]. Afin de détecter les patients hyperparasitémiques et d'améliorer le suivi de la clairance parasitaire, il est important de s'assurer que le calcul de la parasitémie à partir d'un nombre moyen de 6 000 ou de 8 000 leucocytes/mm^3^ n'induit pas une variation importante de la densité parasitaire.

La présente étude se fixe donc comme objectif de déterminer l'impact du nombre de leucocytes sur le calcul de la densité parasitaire des plasmodies en utilisant le nombre réel de leucocytes du patient comparé au nombre de leucocytes moyen proposé par l'OMS et par le PNLP du Bénin.

## Cadre et Méthode D’étude

L’étude a été réalisée dans les formations sanitaires de deux villes du Bénin : l'hôpital de zone de Klouékanmey situé au Sud du Bénin et le centre de santé de Djougou situé au Nord.

La commune de Klouékanmey est une ville du département du Couffo. Sa superficie est de 374 km^2^. Elle comptait 128 597 habitants selon le recensement de 2013 (RGPH-4) [6]. Son climat est de type subéquatorial humide et chaud, avec deux saisons pluvieuses et deux saisons sèches. La pluviométrie annuelle varie entre 900 et 1 200 mm. Ses coordonnées géographiques sont 6°58’49’’ Nord et 1°50’32” Est.

Quant à Djougou, elle est située au Nord-ouest du Bénin dans le département de la Donga, aux portes du massif de l'Atacora. Ses coordonnées géographiques sont 9°42’00’’ Nord et 1°40’00’’ Est. Elle hébergeait environ 267 812 habitants en 2013 [7]. Son climat est relativement sec. La température moyenne à Djougou est de 27,2 °C. La pluviométrie annuelle est de 709,8 mm.

L’étude est de type transversale, descriptive. La population d’étude était constituée à partir d'un recrutement exhaustif des enfants reçus en consultation chez qui le diagnostic clinique de paludisme simple à *Plasmodium falciparum* était suspecté.

Ont été inclus, les enfants âgés de 6 mois à 59 mois pesant au moins 5 kg, ayant une température axillaire ≥ 37,5 °C au moment de la consultation ou un antécédent de fièvre au cours des 24 dernières heures. L'obtention du consentement éclairé des parents ou du tuteur de l'enfant était obligatoire.

Les critères de non-inclusion étaient la présence d'au moins un signe de gravité du paludisme, des signes de malnutrition sévère ou un état fébrile lié à des pathologies infectieuses sous-jacentes autres que le paludisme ainsi qu'un biparasitisme (*P. falciparum* associé à *P. malariae,* combinaison la plus fréquente au Bénin).

Un prélèvement veineux sur l'anticoagulant éthylène diamine tétra-acétique (EDTA) a été effectué sur chaque enfant pour la réalisation d'un hémogramme. Selon le site, différents automates d'hématologie cellulaire ont été utilisés : le « STEL3 » de LINEAR CHEMICALS au centre de santé de Djougou et l’« ABX MICROS 60 » de HORIBA MEDICAL à l'hôpital de zone de Klouékanmey. Après une épreuve de répétabilité et de reproductibilité, chacun des deux automates a été calibré et les mêmes échantillons de contrôles internes de qualité ont été analysés avec chacun de ces appareils. Chaque échantillon des patients a été analysé sur l'un des deux automates en fonction du site de recrutement. Les résultats de chacun des paramètres obtenus sur les deux appareils étaient semblables.

La goutte épaisse et le frottis sanguin ont été confectionnés à partir du sang capillaire de chaque patient sur une lame porte-objet selon la procédure standardisée. Pour toute goutte épaisse positive, la densité parasitaire a été calculée en comptant le nombre de leucocytes et de parasites tandis que le frottis sanguin a permis l'identification de l'espèce du *Plasmodium* en cause. Deux lames de contrôle de qualité, l'une positive et l'autre négative pour la GE, ont été insérées entre les lames pour le contrôle des résultats. Chaque lame a été examinée par deux techniciens expérimentés. En cas de discordance entre les deux résultats, une troisième lecture de la lame était faite.

Sur le plan éthique, le consentement éclairé des parents ou tuteurs des enfants inclus a été obtenu. Ils ont donné leur accord verbal puis posé leur signature sur le formulaire de consentement éclairé. Les propos recueillis lors de l'entretien n'ont pas été divulgués et les noms des participants ont été remplacés par des codes lors du recueil des données. Les données collectées ont été saisies et analysées dans le logiciel Excel. La comparaison des données qualitatives a été réalisée avec le test de khi 2 avec un intervalle de confiance égal à 95 %. La valeur P < 0,05 a été considérée comme significative. Les résultats ont été essentiellement présentés sous forme de moyenne ou de médiane.

## Résultats

Au total, 476 enfants ont été inclus dont 196 enfants issus de l'hôpital de zone de Klouékanmey et 280 du centre de santé de Djougou. La population d’étude était à prédominance masculine (59 %) avec un sex-ratio de 1,4. La répartition des enfants en fonction de l’âge montrait que 433 enfants (90 %) étaient âgés de 11 à 59 mois. Le nombre moyen de leucocytes retrouvé chez les enfants était 11 700 leucocytes/mm^3^ avec des extrêmes de 3 000 à 36 200 leucocytes/ mm^3^. La goutte épaisse était positive chez 313 enfants soit une prévalence de 65,8 % avec un taux de positivité à 62,1 % à Djougou (174 enfants) et de 70,9 % à Klouékanmey (139 enfants).

Sur les 313 enfants ayant une goutte épaisse positive, le nombre de leucocytes était en moyenne de 11 500 leucocytes/mm^3^. Parmi eux, 205 enfants avaient une anomalie du nombre de globules blancs, soit 17 cas de leucopénie (5,4 %) et 188 cas d'hyperleucocytose (60,1 %).

Avec la formule du PNLP, la parasitémie moyenne était de 47 943 trophozoïtes/µl avec des extrêmes allant de 2 020 à 801 980 trophozoïtes/µl. Avec la formule de l'OMS, la parasitémie moyenne était de 63 936 trophozoïtes/µl (2693-1 069 307). Enfin, en utilisant le nombre de leucocytes réels, la parasitémie moyenne était de 92 290 trophozoïtes/µl avec un minimum à 2 300 et un maximum à 2 371 456 trophozoïtes/μl (Tableau I).

**Tableau I:** Comparaison des moyennes des densités parasitaires en fonction du nombre de leucocytes utilisés Comparison of the means of the parasite density in relation to number of white blood cells used

	DP minimale	DP moyenne	DP maximale
**DP 6 000 (PNLP)**	2 020	47 943	801 980
**DP 8 000 (OMS)**	2 693	63 936	1 069307
**DP GB réelle**	2 300	92 290	2 371 456

DP : Densité parasitaire; GB : Globules blancs Source : Données de terrain

La comparaison des moyennes des densités parasitaires montre une différence significative entre les trois méthodes. En utilisant la formule du PNLP-Bénin, 71 enfants avaient une parasitémie inférieure à 2 000 trophozoïtes/µl et 13 patients, une DP supérieure à 200 000 trophozoïtes/µl. En utilisant la formule de l'OMS, 69 patients avaient une densité parasitaire inférieure à 2 000 trophozoïtes/µl et 25 patients, une parasitémie supérieure à 200 000 trophozoïtes/µl. Enfin, lorsque le nombre réel de globules blancs (GB) était utilisé, 64 enfants avaient une densité parasitaire inférieure à 2 000 trophozoïtes/µl et 35 patients, une parasitémie supérieure à 200 000 trophozoite/µl (Tableaux II et III).

**Tableau II:** Répartition des patients selon la parasitémie Distribution of patients according to parasitaemia

	1 à 2000	2 000 à 200 000	> 200 000	Total	Moyenne
**DP 6 000 (PNLP)**	71	229	13	313	47 943
**DP 8 000 (OMS)**	69	219	25	313	63 936
**DP GB réelle**	64	214	35	313	92 290

DP : Densité parasitaire; GB : Globules blancs Source : Données de terrain

**Tableau III:** Pourcentage de sous-estimation et de surestimation des DP 6 000 et DP 8 000 par rapport aux DP GB réelles Percentage of under- and overestimation of parasite density with 6 000 and 8 000 leukocytes/mm^3^ versus real white blood cells count

	% de sous-estimation	% de surestimation	% d’égalité avec la DP GB réelle
**DP 6 000**	60	6	34
**DP 8 000**	49	15	36

DP : Densité parasitaire; GB : Globules blancs Source : Données de terrain

## Discussion

Un tiers des enfants inclus (163/476) sur la base d'une suspicion clinique de paludisme (fièvre) ne présentaient pas une goutte épaisse positive. Ce qui justifie l'intérêt de la confirmation du diagnostic avant tout traitement. Le nombre de leucocytes à prendre en compte pour le calcul de la DP du paludisme a fait l'objet de nombreux travaux aboutissant à des résultats controversés [3]. La valeur moyenne du nombre de leucocytes retrouvée au cours de notre étude était 11 517 leucocytes/mm^3^ avec une médiane de 11 080 leucocytes/mm^3^ de sang. Ce résultat est supérieur aux nombres de leucocytes standards 6 000 et 8 000 leucocytes/mm^3^ respectivement recommandés par le PNLP du Bénin et l'OMS. Par ailleurs, la leucopénie fréquemment décrite dans la littérature au cours du paludisme [4] n’était retrouvée que dans 5,4 % des cas au cours de notre étude.

Les moyennes des densités parasitaires obtenues en utilisant le nombre de leucocytes présumé du PNLP-Bénin et de l'OMS étaient différentes de celle estimée par la numération leucocytaire réelle. L'application des formules de la densité parasitaire proposées par l'OMS et le PNLP-Bénin induit une importante sous-estimation de la densité parasitaire avec un impact sur le diagnostic et la prise en charge des cas de paludisme grave hyperpa-rasitémique, et elle influe négativement sur la surveillance de la clairance parasitaire. Toutefois, il est nécessaire de préciser l'importance relative des densités plasmodiales car la corrélation entre la clinique et la densité parasitaire reste assez faible. L'intérêt d'une densité parasitaire exacte se retrouve dans les études *in vivo* d'efficacité des antipaludiques.

## Conclusion

Les densités plasmodiales estimées respectivement selon les formules du PNLP du Bénin et de l'OMS à partir d'un nombre de leucocytes à 6 000/µl et 8 000/µl sont généralement sous-estimées par rapport à la parasitémie obtenue en utilisant le nombre réel de globules blancs. Il serait donc utile de reprendre cette étude à grande échelle sur tout le territoire béninois afin de proposer la valeur de la leucocytémie la plus appropriée à utiliser pour estimer la parasitémie dans notre contexte de paludisme holoendémique.

## Éthique

La clairance éthique n'a pas été obtenue pour cette étude. Comme mentionné dans la méthodologie, « le consentement éclairé des parents ou tuteurs des enfants inclus a été obtenu. Ils ont donné leur accord verbal, puis posé leur signature sur le formulaire de consentement éclairé ».

## Contribution des Auteurs

T. Baglo et A. Sianou ont élaboré le protocole d’étude. L. Zohoun et A. Sianou ont colligé et analysé les données sur le terrain.

T. Baglo et A. Zohoun ont réalisé la validation biologique des résultats de l'hémogramme et de la goutte épaisse et rédigé le manuscrit.

D. Kindé Gazard a relu et validé le manuscrit en apportant une contribution substantielle à l'article.

Tous les auteurs ont lu et approuvé la version finale du manuscrit.

## Remerciements

Notre gratitude s'adresse au Programme national de lutte contre le paludisme du Bénin.

## Liens D'intérêt

Les auteurs ne déclarent aucun conflit d'intérêt.
